# High-resolution computed tomography scan dataset of lower Mount Simon Sandstone samples from the Illinois Basin

**DOI:** 10.1016/j.dib.2024.110643

**Published:** 2024-06-21

**Authors:** Magdalena Gill, Mathias Pohl, Sarah Brown, Karl Jarvis, Dustin Crandall

**Affiliations:** a National Energy Technology Laboratory, 3610 Collins Ferry Road, Morgantown, WV 26505, USA; b NETL Support Contractor, 3610 Collins Ferry Road, Morgantown, WV 26505, USA

**Keywords:** Carbon storage, Reservoir characterization, Digital petrography, CT, Porosity, Permeability, CCS

## Abstract

This dataset encompasses high-resolution computed tomography scans of small samples of the lower Mount Simon Sandstone from the subsurface of the Illinois Basin. Samples were collected as part of various geological carbon storage characterization efforts and publications focusing on the Mount Simon as a storage reservoir, with scanning performed at the National Energy Technology Laboratory. Thirty-seven three-dimensional (3D) volumes at various resolutions are described and presented as a resource that illustrates the pore and grain size distributions, as well as other petrographic characteristics. This high-quality, fine resolution, 3D image dataset of an important carbon storage target rock formation can be utilized by researchers as a training dataset for machine learning algorithms and for further reservoir characterizations.

Specifications Table

**Table:** 

Subject	Geology; Renewable Energy, Environment
Specific subject area	Computed Tomography Imaging of Geological Samples; Digital Rocks
Data format	CT image data, supporting text and image files
Type of data	Image stacks in .tiff format; core photographs in .jpg format, .txt files, and .png images with scan parameter metadata
Data collection	Samples of basal Mount Simon Sandstone from subsurface well cores in the Illinois Basin in the United States were imaged with resolutions ranging from 0.68 to 14.8 µm. Scans were obtained using a NorthStar M5000 Industrial Computed Tomography scanner and a Zeiss Versa XRM-400 CT scanner. Some samples were dry, while others were exposed to brine, CO_2_/brine or scCO_2_ prior to scanning, as noted in data descriptions. Scans were processed and scale information was added using Fiji/ImageJ. All available scans with sufficient metadata are included in the database.
Data source location	Various stratigraphic and monitoring wells penetrating the lower Mount Simon Sandstone in the Illinois Basin, including Verification Well #1 (39°52′47.23″N, 88°53′36.21″W) and Verification Well#2 (39°53′32.07″N, 88°53′32.82″W). Well names and locations are noted where possible. Scanning took place at the National Energy Technology Laboratory in Morgantown, West Virginia, United States of America
Data accessibility	Repository name: EDX: NETL's Energy Data Exchange Data identification number: doi:10.18141/2326948 Direct URL to data: https://edx.netl.doe.gov/dataset/mt-simon-sandstone-high-resolution-ct

## 1 Value of the Data

• Dataset represents a large assemblage of high-resolution digital rock data on the Illinois Basin Mount Simon Sandstone, a high-priority target of multiple ongoing geologic carbon sequestration efforts in the United States of America.
• High resolution 3D Computed Tomography images are a source of information on porosity and permeability in a format suitable for a variety of research or modeling needs.
• Dataset can be a source of other advanced petrographic and diagenetic characteristic classifications, such as mineral composition, sediment maturity, and degree of cementation.
• This is a valuable resource to researchers interested in characterizing CO_2_ storage reservoirs, testing petrophysical relationships, and improving the information used to generate reservoir models for geological carbon storage.
• The range of image resolutions provides a training ground for machine learning based analysis to help improve such algorithms.

## 2 Background

The data gathering effort [1] was spurred by a desire to characterize and understand reservoir properties and heterogeneity of the Mount Simon Sandstone at a pore-to-core scale, allowing for the upscaling of acquired data and their application to regional field trends and relationships. The samples were sourced from a variety of stratigraphic characterization wells within the Illinois Basin that penetrated the Mount Simon Sandstone, including from the basal portion of the formation. They were then scanned with varying acquisition parameters at the National Energy Technology Laboratory, in Morgantown, West Virginia. The computed tomography scanning and core flow facility was built to non-destructively evaluate rock properties from millimeter to micron resolutions [2]. The equipment in this facility has been used to investigate a variety of fluid interactions in the Mount Simon Sandstone, including the trapping of CO_2_ droplets in pore spaces [3] and geomechanical alterations due to the flow of carbonated brines [4].

## 3 Data Description

The basal portion of the Mount Simon Sandstone represents some of the oldest (Cambrian) strata in the Illinois Basin and serves as a potential injection zone for multiple geological carbon storage projects [5,6]. The Mount Simon is a fine to coarse-grained, poorly sorted, quartz-rich sandstone with varying arkosic regions. Subdivided into lower, middle, and upper sections, the Mount Simon shows transgressive terrestrial to shallow marine sequences [7]. The Mount Simon is regionally extensive, greater than 457 m thick in portions, is overlain by low permeability sealing formations, and is over 1,524 m deep, making it a viable geologic carbon storage reservoir in the Midwest of the United States [8].

**Fig. 1:**
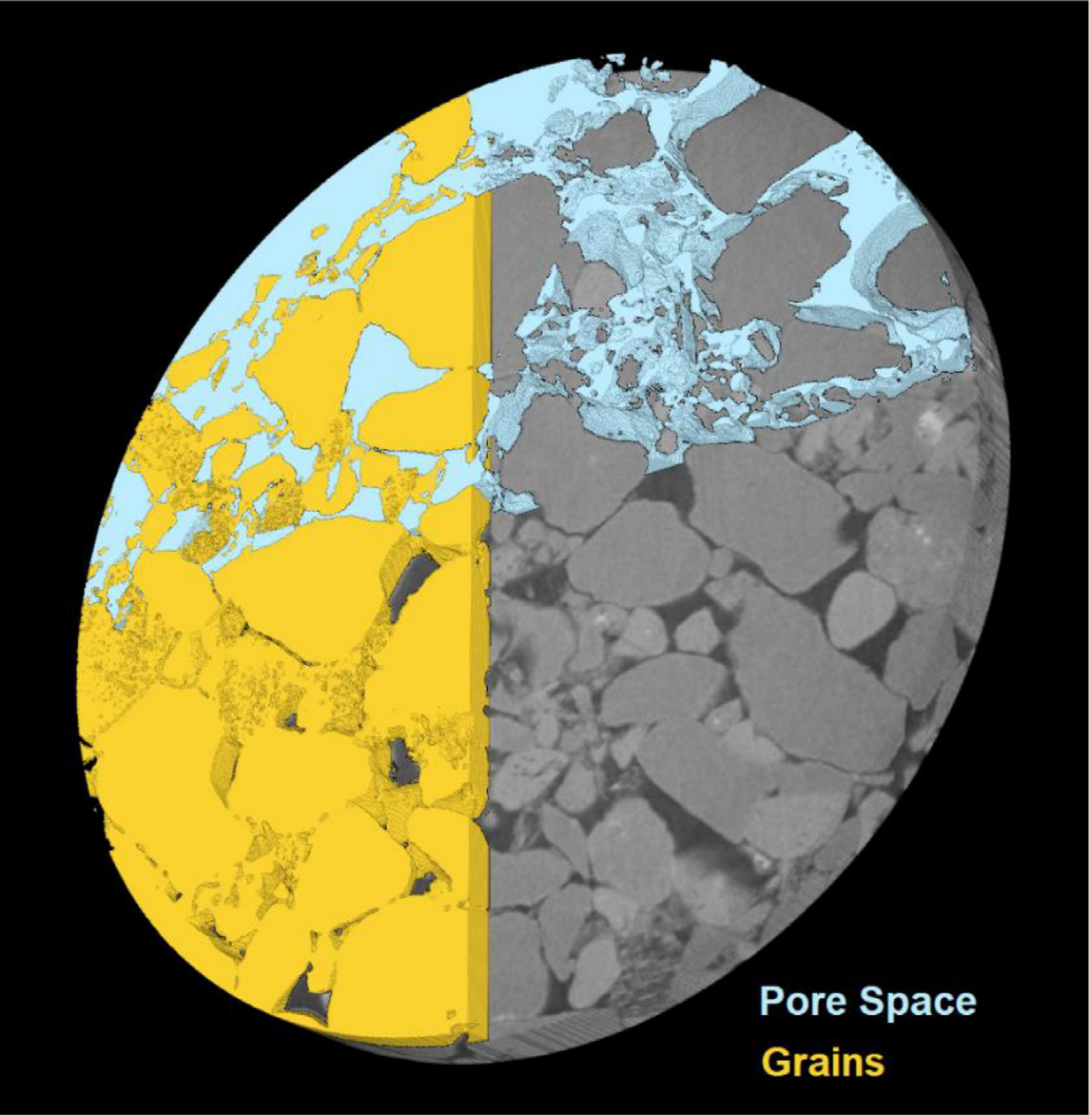
Segmentation: example of pore space and matrix grain isolation in a 2.1 mm diameter digital representation of scan 2-Mt.Simon_6986ft_10x. Pore space is shown in blue, and matrix grains in yellow, with original computed tomography data seen in the background in grayscale.

**Fig. 2:**
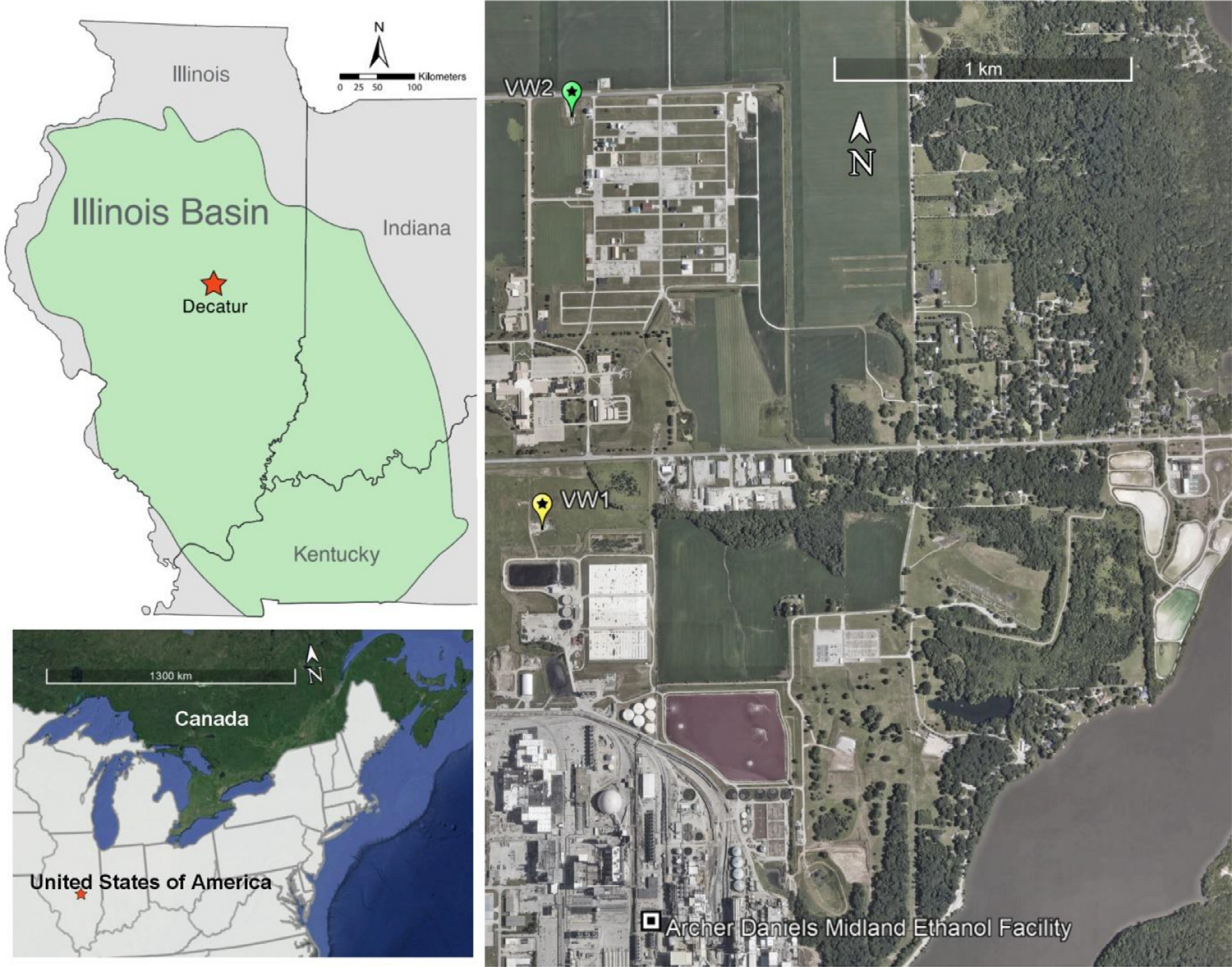
Illinois Basin – Decatur Project location (left) and study site map (right) with location of Verification Well #1 (VW1) and Verification Well #2 (VW2) in Decatur, Illinois, United States [9].

The Mount Simon samples scanned and described (Fig. 1) here are primarily from Verification Well #1 (39°52′47.23′′N, 88°53′36.21′′W) and Verification Well #2 (39°53′32.07′′N, 88°53′32.82′′W) (Fig. 2) drilled as part of the Illinois Basin Decatur Project led by the Illinois State Geological Survey with numerous partners and partially funded by the U.S. Department of Energy. These verification wells were added to this early geological carbon storage site to improve the understanding of plume migration and post-injection site behavior, as described in the NETL Carbon Sequestration Atlas (https://netl.doe.gov/coal/carbon-storage/atlas). Portions of these cores were made available to researchers investigating CO_2_ rock interactions at this location through the Energy Frontier Research Consortium GSCO2, which was in operation from 2014 to 2018 (https://science.osti.gov/bes/efrc/Old-Centers/GSCO2).

**Fig. 3:**
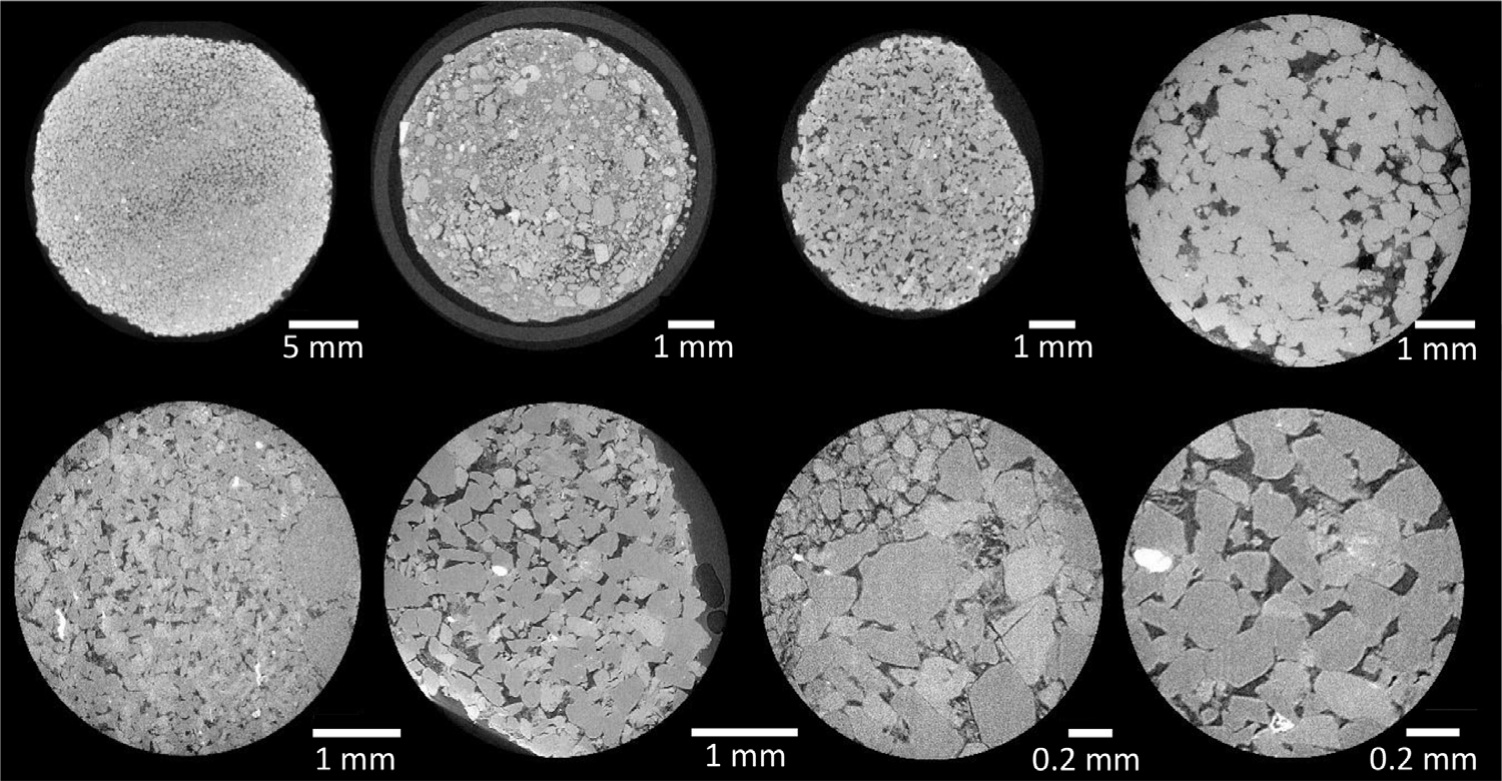
Representative computed tomography slices from a selection of available scans. From left to right: Top row from: 1-Mt.Simon_6986ft_Dry_IndCT, 1-Mt.Simon_VW1_6919.3-6926.6ft_M70, 1-Mt.Simon_6927ft_DryScan_M70, Mt.Simon_VW2_4x_7054.5; and Bottom row: 8-Mt.Simon_VW1_6919.3-6926.6ft_4x_AreaB, 12-Mt.Simon_VW1_6919.3-6926.6ft_Epoxy_4x, 2-Mt.Simon_VW1_6919.3-6926.6ft_10x, 14-Mt.Simon_VW1_6919.3-6926.6ft_Epoxy_10x.

The dataset consists of 37 reconstructed computed tomography scans (Fig. 3) and associated metadata and is housed on NETL's Energy Data eXchange (EDX) [1]. All data are organized by well location (when available) and depth from which samples were obtained in the subsurface and sorted into six scan groups or folders. Most scan groups have multiple scans for a single depth, encompassing different experiments; therefore, scans have been numbered within each folder for organizational purposes. A summary of all the provided data can be found in Table 1: Compilation of all scans and folders (Scan Group) along with file and scan parameter information Table 1. The computed tomography (CT) scanners used for these efforts are NETL's North Star Imagining M-5000 Industrial CT scanner (Industrial CT) and NETL's Zeiss Xradia Versa Micro-CT Scanner (Micro-CT) [1].

**Table 1:** Compilation of all scans and folders (Scan Group) along with file and scan parameter information.

File Name	Scan Group	Scanner	Lens	Resolution (µm)	File Size (GB)	Fluids/Scan Condition
Mt.Simon_6699.6ft_Dry_IndCT	**Mt.Simon_6699.6ft**	Ind CT	n/a	14.8	14.2	Dry
1-Mt.Simon_6927ft_a_DryScan_M70	**Mt.Simon_6927ft**	Micro CT	M70	4.0	5.1	Dry
2-Mt.Simon_6927ft_a_DryScan_4x	**Mt.Simon_6927ft**	Micro CT	4x	1.7	14.9	Dry
1-Mt.Simon_6927ft_CO2Brine_4x	**Mt.Simon_6927ft**	Micro CT	4x	2.3	14.9	CO_2_ Sat Brine
2-Mt.Simon_6927ft_CO2Brine_4x_AreaA	**Mt.Simon_6927ft**	Micro CT	4x	2.3	14.9	CO_2_ Sat Brine
3-Mt.Simon_6927ft_CO2Brine_4x_AreaB	**Mt.Simon_6927ft**	Micro CT	4x	2.3	14.9	CO_2_ Sat Brine
4-Mt.Simon_6927ft_CO2Brine_4x_AreaC	**Mt.Simon_6927ft**	Micro CT	4x	2.3	14.9	CO_2_ Sat Brine
1-Mt.Simon_6986ft_Dry_IndCT	**Mt.Simon_6986ft**	Ind CT	n/a	13.9	4.3	Dry
2-Mt.Simon_6986ft_10x	**Mt.Simon_6986ft**	Micro CT	10x	1.1	14.7	Trace brine
1-Mt.Simon_VW1_6919.3-6926.6ft_M70	**Mt.Simon_VW1_6919.3-6926.6ft**	Micro CT	M70	4.0	15.2	Dry
2-Mt.Simon_VW1_6919.3-6926.6ft_10x	**Mt.Simon_VW1_6919.3-6926.6ft**	Micro CT	10x	0.8	14.5	Dry
3-Mt.Simon_VW1_6919.3-6926.6ft_M70	**Mt.Simon_VW1_6919.3-6926.6ft**	Micro CT	M70	4.0	12.0	Dry
4-Mt.Simon_VW1_6919.3-6926.6ft_4x	**Mt.Simon_VW1_6919.3-6926.6ft**	Micro CT	4x	2.4	14.9	Dry
5-Mt.Simon_VW1_6919.3-6926.6ft_10x	**Mt.Simon_VW1_6919.3-6926.6ft**	Micro CT	10x	0.7	14.5	Dry
6-Mt.Simon_VW1_6919.3-6926.6ft_4x_AreaA	**Mt.Simon_VW1_6919.3-6926.6ft**	Micro CT	4x	2.6	14.8	Dry
7-Mt.Simon_VW1_6919.3-6926.6ft_4x_AreaB	**Mt.Simon_VW1_6919.3-6926.6ft**	Micro CT	4x	2.0	14.9	Dry
8-Mt.Simon_VW1_6919.3-6926.6ft_4x_AreaB	**Mt.Simon_VW1_6919.3-6926.6ft**	Micro CT	4x	2.0	14.9	Dry
9-Mt.Simon_VW1_6919.3-6926.6ft_4x_AreaB	**Mt.Simon_VW1_6919.3-6926.6ft**	Micro CT	4x	2.0	14.9	Dry
10-Mt.Simon_VW1_6919.3-6926.6ft_4x_AreaC	**Mt.Simon_VW1_6919.3-6926.6ft**	Micro CT	4x	2.6	14.9	Dry
11-Mt.Simon_VW1_6919.3-6926.6ft_4x_AreaD	**Mt.Simon_VW1_6919.3-6926.6ft**	Micro CT	4x	1.0	14.5	Dry
12-Mt.Simon_VW1_6919.3-6926.6ft_Epoxy_4x	**Mt.Simon_VW1_6919.3-6926.6ft**	Micro CT	4x	1.7	14.8	Epoxied
13-Mt.Simon_VW1_6919.3-6926.6ft_Epoxy_M70	**Mt.Simon_VW1_6919.3-6926.6ft**	Micro CT	M70	6.6	4.1	Epoxied
14-Mt.Simon_VW1_6919.3-6926.6ft_Epoxy_10x	**Mt.Simon_VW1_6919.3-6926.6ft**	Micro CT	10x	0.7	14.4	Epoxied
1-Mt.Simon_VW1_6926ft_Brine_M70	**Mt.Simon_VW1_6926ft**	Micro CT	M70	6.4	5.8	Brine
2-Mt.Simon_VW1_6926ft_Brine_4x	**Mt.Simon_VW1_6926ft**	Micro CT	4x	2.2	15.0	Brine
3-Mt.Simon_VW1_6926ft_Brine_M70	**Mt.Simon_VW1_6926ft**	Micro CT	M70	6.4	4.2	Brine
4-Mt.Simon_VW1_6926ft_CO2Brine_M70	**Mt.Simon_VW1_6926ft**	Micro CT	M70	6.4	5.8	CO_2_ Sat Brine
5-Mt.Simon_VW1_6926ft_CO2Brine_4x	**Mt.Simon_VW1_6926ft**	Micro CT	4x	2.2	14.9	CO_2_ Sat Brine
6-Mt.Simon_VW1_6926ft_CO2_into_CO2Brine_4x	**Mt.Simon_VW1_6926ft**	Micro CT	4x	2.2	14.9	CO_2_ Sat Brine
7-Mt.Simon_VW1_6926ft_scCO2_into_scCO2Brine_M70	**Mt.Simon_VW1_6926ft**	Micro CT	M70	6.4	4.1	SuperCrit CO_2_
8-Mt.Simon_VW1_6926ft_scCO2_into_scCO2Brine_4x	**Mt.Simon_VW1_6926ft**	Micro CT	4x	2.2	14.9	SuperCrit CO_2_
1-Mt.Simon_VW2_4x_7055.5	**Mt.Simon_VW2_7030.5_7055.5ft**	Micro CT	4x	2.0	14.9	Dry
2-Mt.Simon_VW2_4x_7055.5	**Mt.Simon_VW2_7030.5_7055.5ft**	Micro CT	4x	2.0	14.9	Dry
Mt.Simon_VW2_4x_7038.5	**Mt.Simon_VW2_7030.5_7055.5ft**	Micro CT	4x	2.6	14.9	Dry
Mt.Simon_VW2_4x_7030.5	**Mt.Simon_VW2_7030.5_7055.5ft**	Micro CT	4x	2.6	14.9	Dry
Mt.Simon_VW2_4x_7054.5	**Mt.Simon_VW2_7030.5_7055.5ft**	Micro CT	4x	2.8	14.8	Dry
Mt.Simon_VW2_4x_7033.6	**Mt.Simon_VW2_7030.5_7055.5ft**	Micro CT	4x	2.8	14.9	Dry

Folder: ***Mt.Simon_6699.6ft*** contains one scan of a sample from a depth of 6,699.6 ft:

**Mt.Simon_6699.6ft_Dry_IndCT** was obtained with the Industrial CT scanner in a dry condition at a resolution of 14.8 µm per pixel.

The file is a 16-bit .tif stack of images. A single cross-sectional image from the middle of the scan (**Mt.Simon_6699.6ft_Dry_IndCT_slice1000.jpg**) was saved in .jpg format for quick reference. The text file **ScanDetails.txt** contains additional scanning parameters.

Folder: ***Mt.Simon_6927ft*** contains six scans of samples from a depth of 6,927 ft:

**1-Mt.Simon_6927ft_DryScan_M70** was obtained with the Micro-CT using the M70 optic in a dry condition at a resolution of 4.0 µm per pixel.

The file is a 16-bit .tif stack of images. A single cross-sectional image from the middle of the scan (**1-Mt.Simon_6927ft_DryScan_M70_slice1500.jpg**) was saved in .jpg format for quick reference. The file **1-Mt.Simon_6927ft_DryScan_M70_Recipe.png** contains additional scanning parameters.

**2-Mt.Simon_6927ft_DryScan_4x** was obtained with the Micro-CT using the 4x optic in a dry condition at a resolution of 1.7 µm per pixel.

The file is a 16-bit .tif stack of images. A single cross-sectional image from the middle of the scan (**2-Mt.Simon_6927ft_DryScan_4x_slice1000.jpg**) was saved in .jpg format for quick reference. The file **2-Mt.Simon_6927ft_a_DryScan_4x_Recipe.png** contains additional scanning parameters.

**1-Mt.Simon_6927ft_CO2Brine_4x** was obtained with the Micro-CT using the 4x optic at a resolution of 2.3 µm per pixel on a sample injected with CO_2_-saturated brine during the experiment.

The file is a 16-bit .tif stack of images. A single cross-sectional image from the middle of the scan (**1-Mt.Simon_6927ft_CO2Brine_4x_slice1000.jpg**) was saved in .jpg format for quick reference. The file **1-Mt.Simon_6927ft_CO2Brine_4x_Recipe.png** contains additional scanning parameters.

**2-Mt.Simon_6927ft_CO2Brine_4x_AreaA** was obtained with the Micro-CT using the 4x optic at a resolution of 2.3 µm per pixel on a sample injected with CO_2_-saturated brine during the experiment.

The file is a 16-bit .tif stack of images. A single cross-sectional image from the middle of the scan (**2-Mt.Simon_6927ft_CO2Brine_4x_AreaA_slice1000.jpg**) was saved in .jpg format for quick reference. The file **2-Mt.Simon_6927ft_CO2Brine_4x_AreaA_Recipe.png** contains additional scanning parameters.

**3-Mt.Simon_6927ft_CO2Brine_4x_AreaB** was obtained with the Micro-CT using the 4x optic at a resolution of 2.3 µm per pixel on a sample injected with CO_2_-saturated brine during the experiment.

The file is a 16-bit .tif stack of images. A single cross-sectional image from the middle of the scan (**3-Mt.Simon_6927ft_CO2Brine_4x_AreaB_slice1000.jpg**) was saved in .jpg format for quick reference. The file **3-Mt.Simon_6927ft_CO2Brine_4x_AreaB_Recipe.png** contains additional scanning parameters.

**4-Mt.Simon_6927ft_CO2Brine_4x_AreaC** was obtained with the Micro-CT using the 4x optic at a resolution of 2.3 µm per pixel on a sample injected with CO_2_-saturated brine during the experiment.

The file is a 16-bit .tif stack of images. A single cross-sectional image from the middle of the scan (**4-Mt.Simon_6927ft_CO2Brine_4x_AreaC_slice500.jpg**) was saved in .jpg format for quick reference. The file **4-Mt.Simon_6927ft_CO2Brine_4x_AreaC_Recipe.png** contains additional scanning parameters.

Folder: ***Mt.Simon_6986ft*** contains two scans of samples from a depth of 6,986 ft:

**1_Mt.Simon_6986ft_Dry_IndCT** was obtained with the Industrial CT scanner in a dry condition at a resolution of 13.9 µm per pixel.

The file is a 16-bit .tif stack of images. A single cross-sectional image (**Mt.Simon_6986ft_Dry_IndCT_slice400.jpg**) was saved in .jpg format for quick reference. The text file **ScanDetails.txt** contains additional scanning parameters.

**2-Mt.Simon_6986ft_10x** was obtained with the Micro-CT using the 10x optic at a resolution of 1.1 µm per pixel on a sample with trace brine in the pore space.

The file is a 16-bit .tif stack of images. A single cross-sectional image from the middle of the scan (**2-Mt.Simon_6986ft_10x_slice1000.jpg**) was saved in .jpg format for quick reference. The file **2-Mt.Simon_6986ft_10x_Recipe.png** contains additional scanning parameters.

Folder: ***Mt.Simon_VW1_6919.3-6926.6ft*** contains 14 scans of samples from a depth range of 6,919.3 to 6,926.6 ft from Verification Well #1:

**1-Mt.Simon_VW1_6919.3-6926.6ft_M70** was obtained with the Micro-CT using the M70 optic in a dry condition at a resolution of 4.0 µm per pixel.

The file is a 16-bit .tif stack of images. A single cross-sectional image from the middle of the scan (**1-Mt.Simon_VW1_6919.3-6926.6ft_M70_slice1000.jpg**) was saved in .jpg format for quick reference. The file **1-Mt.Simon_VW1_6919.3-6926.6ft_M70_Recipe.png** contains additional scanning parameters.

**2-Mt.Simon_VW1_6919.3-6926.6ft_10x** was obtained with the Micro-CT using the 10x optic in a dry condition at a resolution of 0.8 µm per pixel.

The file is a 16-bit .tif stack of images. A single cross-sectional image from the middle of the scan (**2-Mt.Simon_VW1_6919.3-6926.6ft_10x_slice1000.jpg**) was saved in .jpg format for quick reference. The file **2-Mt.Simon_VW1_6919.3-6926.6ft_10x_Recipe.png** contains additional scanning parameters.

**3-Mt.Simon_VW1_6919.3-6926.6ft_M70** was obtained with the Micro-CT using the M70 optic in a dry condition at a resolution of 4.0 µm per pixel.

The file is a 16-bit .tif stack of images. A single cross-sectional image from the middle of the scan (**3-Mt.Simon_VW1_6919.3-6926.6ft_M70_slice1000.jpg**) was saved in .jpg format for quick reference. The file **3-Mt.Simon_VW1_6919.3-6926.6ft_M70_Recipe.png** contains additional scanning parameters.

**4-Mt.Simon_VW1_6919.3-6926.6ft_4x** was obtained with the Micro-CT using the 4x optic in a dry condition at a resolution of 2.4 µm per pixel.

The file is a 16-bit .tif stack of images. A single cross-sectional image from the middle of the scan (**4-Mt.Simon_VW1_6919.3-6926.6ft_4x_slice1000.jpg**) was saved in .jpg format for quick reference. The file **4-Mt.Simon_VW1_6919.3-6926.6ft_4x_Recipe.png** contains additional scanning parameters.

**5-Mt.Simon_VW1_6919.3-6926.6ft_10x** was obtained with the Micro-CT using the 10x optic in a dry condition at a resolution of 0.7 µm per pixel.

The file is a 16-bit .tif stack of images. A single cross-sectional image from the middle of the scan (**5-Mt.Simon_VW1_6919.3-6926.6ft_10x_slice1000.jpg**) was saved in .jpg format for quick reference. The file **5-Mt.Simon_VW1_6919.3-6926.6ft_10x_Recipe.png** contains additional scanning parameters.

**6-Mt.Simon_VW1_6919.3-6926.6ft_4x_AreaA** was obtained with the Micro-CT using the 4x optic in a dry condition at a resolution of 2.6 µm per pixel.

The file is a 16-bit .tif stack of images. A single cross-sectional image from the middle of the scan (**6-Mt.Simon_VW1_6919.3-6926.6ft_4x_AreaA_slice1000.jpg**) was saved in .jpg format for quick reference. The file **6-Mt.Simon_VW1_6919.3-6926.6ft_4x_AreaA_Recipe.png** contains additional scanning parameters.

**7-Mt.Simon_VW1_6919.3-6926.6ft_4x_AreaB** was obtained with the Micro-CT using the 4x optic in a dry condition at a resolution of 2.0 µm per pixel.

The file is a 16-bit .tif stack of images. A single cross-sectional image from the middle of the scan (**7-Mt.Simon_VW1_6919.3-6926.6ft_4x_AreaB_slice1000.jpg**) was saved in .jpg format for quick reference. The file **7-Mt.Simon_VW1_6919.3-6926.6ft_4x_AreaB_Recipe.png** contains additional scanning parameters.

**8-Mt.Simon_VW1_6919.3-6926.6ft_4x_AreaB** was obtained with the Micro-CT using the 4x optic in a dry condition at a resolution of 2.0 µm per pixel.

The file is a 16-bit .tif stack of images. A single cross-sectional image from the middle of the scan (**8-Mt.Simon_VW1_6919.3-6926.6ft_4x_AreaB_slice1000.jpg**) was saved in .jpg format for quick reference. The file **8-Mt.Simon_VW1_6919.3-6926.6ft_4x_AreaB_Recipe.png** contains additional scanning parameters.

**9-Mt.Simon_VW1_6919.3-6926.6ft_4x_AreaB** was obtained with the Micro-CT using the 4x optic in a dry condition at a resolution of 2.0 µm per pixel.

The file is a 16-bit .tif stack of images. A single cross-sectional image from the middle of the scan (**9-Mt.Simon_VW1_6919.3-6926.6ft_4x_AreaB_slice1000.jpg**) was saved in .jpg format for quick reference. The file **9-Mt.Simon_VW1_6919.3-6926.6ft_4x_AreaB_Recipe.png** contains additional scanning parameters.

**10-Mt.Simon_VW1_6919.3-6926.6ft_4x_AreaC** was obtained with the Micro-CT using the 4x optic in a dry condition at a resolution of 2.6 µm per pixel.

The file is a 16-bit .tif stack of images. A single cross-sectional image from the middle of the scan (**10-Mt.Simon_VW1_6919.3-6926.6ft_4x_AreaC_slice1000.jpg**) was saved in .jpg format for quick reference. The file **10-Mt.Simon_VW1_6919.3-6926.6ft_4x_AreaC_Recipe.png** contains additional scanning parameters.

**11-Mt.Simon_VW1_6919.3-6926.6ft_4x_AreaD** was obtained with the Micro-CT using the 4x optic in a dry condition at a resolution of 1.0 µm per pixel.

The file is a 16-bit .tif stack of images. A single cross-sectional image from the middle of the scan (**11-Mt.Simon_VW1_6919.3-6926.6ft_4x_AreaD_slice1000.jpg**) was saved in .jpg format for quick reference. The file **11-Mt.Simon_VW1_6919.3-6926.6ft_4x_AreaD_Recipe.png** contains additional scanning parameters.

**12-Mt.Simon_VW1_6919.3-6926.6ft_Epoxy_4x** was obtained with the Micro-CT using the 4x optic in a dry condition at a resolution of 1.7 µm per pixel on an epoxy-stabilized sample.

The file is a 16-bit .tif stack of images. A single cross-sectional image from the middle of the scan (**12-Mt.Simon_VW1_6919.3-6926.6ft_Epoxy_4x_slice1000.jpg**) was saved in .jpg format for quick reference. The file **12-Mt.Simon_VW1_6919.3-6926.6ft_Epoxy_4x_Recipe.png** contains additional scanning parameters.

**13-Mt.Simon_VW1_6919.3-6926.6ft_Epoxy_M70** was obtained with the Micro-CT using the M70 optic in a dry condition at a resolution of 6.6 µm per pixel on an epoxy-stabilized sample.

The file is a 16-bit .tif stack of images. A single cross-sectional image from the middle of the scan (**13-Mt.Simon_VW1_6919.3-6926.6ft_Epoxy_M70_slice1000.jpg**) was saved in .jpg format for quick reference. The file **13-Mt.Simon_VW1_6919.3-6926.6ft_Epoxy_M70_Recipe.png** contains additional scanning parameters.

**14-Mt.Simon_VW1_6919.3-6926.6ft_Epoxy_10x** was obtained with the Micro-CT using the 10x optic in a dry condition at a resolution of 0.7 µm per pixel on an epoxy-stabilized sample.

The file is a 16-bit .tif stack of images. A single cross-sectional image from the middle of the scan (**14-Mt.Simon_VW1_6919.3-6926.6ft_Epoxy_19x_slice1000.jpg**) was saved in .jpg format for quick reference. The file **14-Mt.Simon_VW1_6919.3-6926.6ft_Epoxy_10x_Recipe.png** contains additional scanning parameters.

Folder: ***Mt.Simon_VW1_6926ft*** contains eight scans of samples from a depth of 6,926 ft from Verification Well #1:

**1-Mt.Simon_VW1_6926ft_Brine_M70** was obtained with the Micro-CT using the M70 optic at a resolution of 6.4 µm per pixel on sample that was injected with brine during the experiment.

The file is a 16-bit .tif stack of images. A single cross-sectional image from the middle of the scan (**1-Mt.Simon_VW1_6926ft_Brine_M70_slice1000.jpg**) was saved in .jpg format for quick reference. The file **1-Mt.Simon_VW1_6926ft_Brine_M70_Recipe.png** contains additional scanning parameters.

**2-Mt.Simon_VW1_6926ft_Brine_4x** was obtained with the Micro-CT using the 4x optic at a resolution of 2.2 µm per pixel on sample that was injected with brine during the experiment.

The file is a 16-bit .tif stack of images. A single cross-sectional image from the middle of the scan (**2-Mt.Simon_VW1_6926ft_Brine_4x_slice1000.jpg**) was saved in .jpg format for quick reference. The file **2-Mt.Simon_VW1_6926ft_Brine_4x_Recipe.png** contains additional scanning parameters.

**3-Mt.Simon_VW1_6926ft_Brine_M70** was obtained with the Micro-CT using the M70 optic at a resolution of 6.4 µm per pixel on a sample that was injected with brine during the experiment.

The file is a 16-bit .tif stack of images. A single cross-sectional image from the middle of the scan (**3-Mt.Simon_VW1_6926ft_Brine_M70_slice1000.jpg**) was saved in .jpg format for quick reference. The file **3-Mt.Simon_VW1_6926ft_Brine_M70_Recipe.png** contains additional scanning parameters.

**4-Mt.Simon_VW1_6926ft_CO2Brine_M70** was obtained with the Micro-CT using the M70 optic at a resolution of 6.4 µm per pixel on a sample that was injected with CO_2_-saturated brine during the experiment.

The file is a 16-bit .tif stack of images. A single cross-sectional image from the middle of the scan (**4-Mt.Simon_VW1_6926ft_CO2Brine_M70_slice1000.jpg**) was saved in .jpg format for quick reference. The file **4-Mt.Simon_VW1_6926ft_CO2Brine_M70_Recipe.png** contains additional scanning parameters.

**5-Mt.Simon_VW1_6926ft_CO2Brine_4x** was obtained with the Micro-CT using the 4x optic at a resolution of 2.2 µm per pixel on a sample that was injected with CO_2_-saturated brine during the experiment.

The file is a 16-bit .tif stack of images. A single cross-sectional image from the middle of the scan (**5-Mt.Simon_VW1_6926ft_CO2Brine_4x_slice1000.jpg**) was saved in .jpg format for quick reference. The file **5-Mt.Simon_VW1_6926ft_CO2Brine_4x_Recipe.png** contains additional scanning parameters.

**6-Mt.Simon_VW1_6926ft_CO2_into_CO2Brine_4x** was obtained with the Micro-CT using the 4x optic at a resolution of 2.2 µm per pixel on a sample that was being injected with CO_2_ into a core pre-saturated with CO_2_-saturated brine.

The file is a 16-bit .tif stack of images. A single cross-sectional image from the middle of the scan (**6-Mt.Simon_VW1_6926ft_CO2_into_CO2Brine_4x _slice1000.jpg**) was saved in .jpg format for quick reference. The file **6-Mt.Simon_VW1_6926ft_CO2_into_CO2Brine_4x _Recipe.png** contains additional scanning parameters.

**7-Mt.Simon_VW1_6926ft_scCO2_into_scCO2Brine_M70** was obtained with the Micro-CT using the M70 optic at a resolution of 6.4 µm per pixel on a sample that was being injected with supercritical CO_2_ (scCO_2_) into a core pre-saturated with scCO_2_-saturated brine.

The file is a 16-bit .tif stack of images. A single cross-sectional image from the middle of the scan (**7-Mt.Simon_VW1_6926ft_scCO2_into_scCO2Brine_M70_slice1000.jpg**) was saved in .jpg format for quick reference. The file **7-Mt.Simon_VW1_6926ft_scCO2_into_scCO2Brine_M70_Recipe.png** contains additional scanning parameters.

**8-Mt.Simon_VW1_6926ft_scCO2_into_scCO2Brine_4x** was obtained with the Micro-CT using the 4x optic at a resolution of 2.2 µm per pixel on a sample that was being injected with supercritical CO_2_ (scCO_2_) into a core pre-saturated with scCO_2_-saturated brine.

The file is a 16-bit .tif stack of images. A single cross-sectional image from the middle of the scan (**8-Mt.Simon_VW1_6926ft_scCO2_into_scCO2Brine_4x_slice1000.jpg**) was saved in .jpg format for quick reference. The file **8-Mt.Simon_VW1_6926ft_scCO2_into_scCO2Brine_4x_Recipe.png** contains additional scanning parameters.

Folder: ***Mt.Simon_VW2_7030.5_7055.5ft*** contains six scans of samples from a depth range of 7,030.5-7,055.5 ft from Verification Well #2:

**1-Mt.Simon_VW2_4x_7055.5** was obtained with the Micro-CT using the 4x optic in a dry condition at a resolution of 2.0 µm per pixel.

The file is a 16-bit .tif stack of images. A single cross-sectional image from the middle of the scan (**1-Mt.Simon_VW2_4x_7055.5_slice1000.jpg**) was saved in .jpg format for quick reference. The file **1-Mt.Simon_VW2_4x_7055.5_Recipe.png** contains additional scanning parameters.

**2-Mt.Simon_VW2_4x_7055.5** was obtained with the Micro-CT using the 4x optic in a dry condition at a resolution of 2.0 µm per pixel.

The file is a 16-bit .tif stack of images. A single cross-sectional image from the middle of the scan (**2-Mt.Simon_VW2_4x_7055.5_slice1000.jpg**) was saved in .jpg format for quick reference. The file **2-Mt.Simon_VW2_4x_7055.5_Recipe.png** contains additional scanning parameters.

**Mt.Simon_VW2_4x_7038.5** was obtained with the Micro-CT using the 4x optic in a dry condition at a resolution of 2.6 µm per pixel.

The file is a 16-bit .tif stack of images. A single cross-sectional image from the middle of the scan (**Mt.Simon_VW2_4x_7038.5_slice1000.jpg**) was saved in .jpg format for quick reference. The file **Mt.Simon_VW2_4x_7038.5_Recipe.png** contains additional scanning parameters.

**Mt.Simon_VW2_4x_7030.5** was obtained with the Micro-CT using the 4x optic in a dry condition at a resolution of 2.6 µm per pixel.

The file is a 16-bit .tif stack of images. A single cross-sectional image from the middle of the scan (**Mt.Simon_VW2_4x_7030.5_slice1000.jpg**) was saved in .jpg format for quick reference. The file **Mt.Simon_VW2_4x_7030.5_Recipe.png** contains additional scanning parameters.

**Mt.Simon_VW2_4x_7054.5** was obtained with the Micro-CT using the 4x optic in a dry condition at a resolution of 2.8 µm per pixel.

The file is a 16-bit .tif stack of images. A single cross-sectional image from the middle of the scan (**Mt.Simon_VW2_4x_7054.5_slice1000.jpg**) was saved in .jpg format for quick reference. The file **Mt.Simon_VW2_4x_7054.5_Recipe.png** contains additional scanning parameters.

**Mt.Simon_VW2_4x_7033.6** was obtained with the Micro-CT using the 4x optic in a dry condition at a resolution of 2.8 µm per pixel.

The file is a 16-bit .tif stack of images. A single cross-sectional image from the middle of the scan (**Mt.Simon_VW2_4x_7033.3_slice1000.jpg**) was saved in .jpg format for quick reference. The file **Mt.Simon_VW2_4x_7033.6_Recipe.png** contains additional scanning parameters.

## 4 Experimental Design, Materials and Methods

The Mount Simon dataset consists of two standard core sized samples (nominally ∼2.5 cm in diameter) and an assortment of smaller (<1 cm in diameter) samples numbering nine or more (please refer to the limitations section for a detailed explanation regarding the number of samples versus the number of scans). The total number of scans is 37.

At the time, the lower Mount Simon Sandstone was selected as the primary injection interval due to its large storage potential with porosities of up to 30 % [10]. Samples for this study were subcored from larger competent core sections, identified as the basal Mount Simon Sandstone. Heavily fractured or damaged segments were avoided during selection of subcore locations.

Standard core size samples were prepared by drilling sub-cores 2.54 cm in diameter, with varying lengths: Mt.Simon_6699.6ft_Dry_IndCT (3.6 cm) and Mt.Simon_6986ft_Dry_IndCT (0.74 cm). These were scanned in a dry state at room temperature and pressure in the NorthStar Imaging Industrial M5000 CT-scanner at a resolution of 14.8 µm and 13.9 µm, respectively. The scans were then reconstructed with the proprietary NorthStar efXCT software and exported as 16-bit .tiff image stacks. All image processing, consisting of cropping and scale application, was performed using ImageJ/Fiji [11].

The <1 cm in diameter samples were sub-cored in preparation for scanning in the Zeiss Versa XRM-400 CT scanner (Micro-CT), with a core diameter of 0.65 cm. Due to the friable nature of samples some cores are fragmentary and deviate from a round cross-sectional view. One such sample (Fig. 3), represented in scans 12-Mt.Simon_VW1_6919.3-6926.6ft_Epoxy_4x, 13-Mt.Simon_VW1_6919.3-6926.6ft_Epoxy_M70, 14-Mt.Simon_VW1_6919.3-6926.6ft_Epoxy_10x, was stabilized in Devcon 2 Ton Epoxy, a low viscosity resin with superior pore penetration, to prevent further crumbling. The sample was dipped in premixed epoxy and left to cure at ambient conditions to provide stability on the scanning stage.

Small samples were scanned at one of six experimental conditions: dry, dry epoxy stabilized, brine, CO_2_-saturated brine flow tests, supercritical CO_2_, and a post-flow condition with trace brine present in pore space. The experimental condition for each of the tests can be found in Table 1.

For samples scanned in a dry or a dry epoxy-stabilized state, the sample was placed in the Zeiss Versa XRM-400 CT scanner for scanning at ambient pressure and temperature conditions.

Samples scanned with fluids present were placed in a rubber confining sleeve with spacers at each end. Then the samples were secured inside a custom designed, computed tomography-transparent beryllium core holder. Three Teledyne ISCO pumps were used in the core flooding system maintaining pore and confining pressure [3]. Samples were then injected with brine or CO_2_-saturated brine (denoted respectively as Brine and CO_2_ Sat Brine in Table 1). For samples with supercritical CO_2_ pressure and temperature conditions were elevated to achieve supercritical conditions (14.5 MPa confining pressure, and 47°C). Trace brine is visible in one sample scanned at ambient conditions (2-Mt.Simon_6986ft_10x).

All Micro-CT scans were conducted using one of three optic lenses: the 10x, the 4x, or the M70, depending on desired resolution. The 10x optic allows for the highest resolution scans and was used to obtain scans in the 0.7-1.1 µm resolution range. The 4x optic was used for scans in the 1.0-2.8 µm resolution range, and the M70 for scans with resolutions of 4.0-6.6 µm. For detailed information on which optic and what resolution was achieved for each scan, please refer to Table 1.

All micro-CT scans were reconstructed using proprietary Xradia software and exported as 16-bit .tiff image stacks. All image processing, consisting of cropping and scale application, was performed in ImageJ/Fiji [11].

## Limitations

This data curation effort was undertaken with respect to samples analyzed over a period of several years. The prolonged duration nature of the data acquisition meant some parameters could no longer be verified. Specifically, the exact number of small samples could not be established, as many physical samples have already been returned to their research entities of origin. While multiple scans of the same well and depth exist, it was not always possible to establish whether these encompass various parts of the same physical sample, or whether they relate to multiple physical subcores obtained from the same larger core and correspond to the same depth. All available sample information was included in the naming scheme and data table, including the well and depth from which samples were sourced, the scan type (IndCT, M70, 4x, 10x) and the area scanned when one physical sample was confirmed to have been scanned repeatedly (e.g., AreaA, AreaB).

Additionally, due to the proprietary nature of some experiments, three scan groups do not have a physical well location attached to them. In those cases, known parameters include only the generalized source within the Illinois Basin, and the depth from which samples were obtained.

## Ethics Statement

The authors have adhered to all ethical requirements for publication in Data in Brief and confirm that this dataset does not make use of human subjects, animal experiments, nor does it contain any data collected from social media platforms.

## CRediT authorship contribution statement

**Magdalena Gill:** Data curation, Validation, Writing – original draft. **Mathias Pohl:** Data curation, Validation, Visualization, Writing – review & editing. **Sarah Brown:** Data curation. **Karl Jarvis:** Data curation, Writing – review & editing. **Dustin Crandall:** Conceptualization, Supervision, Project administration, Writing – original draft.

## Data Availability

Mt. Simon Sandstone - High Resolution CT (Original data) (EDX).
